# DNA Suspension Arrays: Silencing Discrete Artifacts for High-Sensitivity Applications

**DOI:** 10.1371/journal.pone.0015476

**Published:** 2010-11-08

**Authors:** Matthew S. Lalonde, Eric J. Arts

**Affiliations:** 1 Department of Biochemistry, Case Western Reserve University, Cleveland, Ohio, United States of America; 2 Division of Infectious Diseases, Department of Medicine, Case Western Reserve University, Cleveland, Ohio, United States of America; Institut Pasteur, France

## Abstract

Detection of low frequency single nucleotide polymorphisms (SNPs) has important implications in early screening for tumorgenesis, genetic disorders and pathogen drug resistance. Nucleic acid arrays are a powerful tool for genome-scale SNP analysis, but detection of low-frequency SNPs in a mixed population on an array is problematic. We demonstrate a model assay for HIV-1 drug resistance mutations, wherein ligase discrimination products are collected on a suspension array. In developing this system, we discovered that signal from multiple polymorphisms was obscured by two discrete hybridization artifacts. Specifically: 1) tethering of unligated probes on the template DNA elicited false signal and 2) unpredictable probe secondary structures impaired probe capture and suppressed legitimate signal from the array. Two sets of oligonucleotides were used to disrupt these structures; one to displace unligated reporter labels from the bead-bound species and another to occupy sequences which interfered with array hybridization. This artifact silencing system resulted in a mean 21-fold increased sensitivity for 29 minority variants of 17 codons in our model assay for mutations most commonly associated with HIV-1 drug resistance. Furthermore, since the artifacts we characterized are not unique to our system, their specific inhibition might improve the quality of data from solid-state microarrays as well as from the growing number of multiple analyte suspension arrays relying on sequence-specific nucleic acid target capture.

## Introduction

Assays for single nucleotide polymorphisms (SNPs) are useful for haplotyping [1], identifying pathogen species [2], and screening for heritable polymorphisms [3]. While many methods are available for detecting *dominant* SNPs, a simple multiplex platform to detect *minority variants* might offer greater information yield and could be used for a broader scope of applications such as detecting SNPs implicated in somatic cell carcinogenesis [4] or identifying drug resistance mutations arising from pathogen or cancer chemotherapy [5].

Suspension array platforms, like that available from Luminex (www.luminexcorp.com), are an attractive means for multiple SNP detection since they allow rapid identification of multiple species in parallel. Target molecules are captured on optically identifiable beads and quantified by a flow fluorimeter, an instrument equivalent to a three-color flow cytometer with dedicated gating for beads in suspension [6]. This system, equivalent to a solid-state array in many capacities, can be used to simultaneously detect multiple proteins [7] or nucleic acids [2] via identifiable fluorescent signatures on beads with a corresponding affinity label.

**Figure 1:**
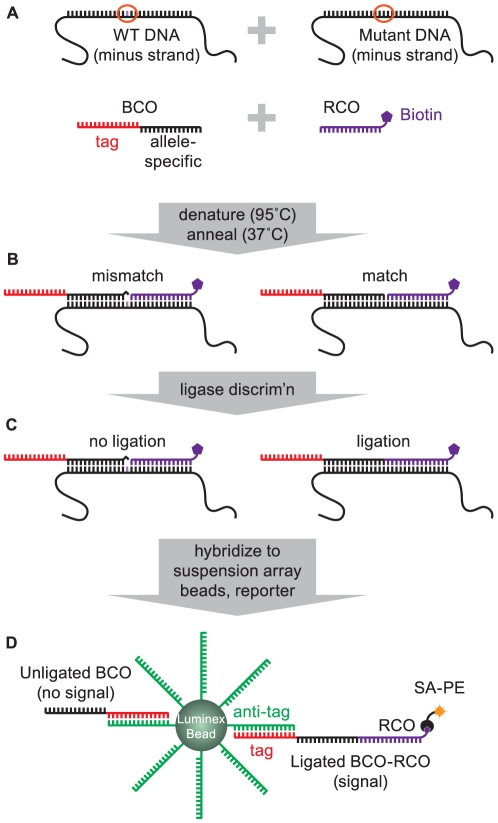
Ligase discrimination and bead capture. (A) Depiction of a 50% mixture of wild-type and mutant template (only the negative strand is shown) in a ligase discrimination reaction query for the mutant SNP. The mutant BCO comprises an allele-specific region (black) with a template-unrelated tag sequence at the 5′-end (red). The RCO is complementary to the mutant template DNA downstream from the SNP of interest and is conjugated to a 3′-biotin. (B) During annealing, a mismatch occurs between the wild-type template and the mutant BCO 3′-end (left) whereas the mutant probes are complemented by the template across the probe junction. (C) A template-dependent DNA ligase joins the mutant probe pair on the mutant DNA, but does not join mutant probes on the wild-type template DNA due to the mismatch close to the probe junction. (D) Ligase discrimination products are hybridized to Luminex beads coupled to anti-tag sequences. Biotin residues on bead-bound RCOs capture a streptavidin-R-phycoerythrin reporter. Detection of bead-associated reporter then reflects the fraction of mutant template in the sample.

For SNP detection in the present study, we use this system to capture and quantify ligase discrimination reaction (LDR) products. LDR is the discriminatory step in an oligonucleotide ligation assay (OLA) and uses a high-fidelity DNA ligase to join two adjacent oligonucleotide probes on a DNA template at a designated mutation [8] (Figure 1A–C). Ligation products are captured via a bead-specific “tag” sequence on the 5′-terminus of the up-stream oligonucleotide (bead capture oligonucleotide; BCOs) and a biotin modification on the 3′-terminus of the downstream oligonucleotide (reporter capture oligonucleotide; RCO; Figure 1A). BCOs and BCO-RCO ligation products are collected on beads conjugated to appropriate “antitag” sequences (the tag complement) and which contain a dual-fluorophore mixture (for identification; Figure 1D). Bead-captured BCO-RCO ligation products are labeled with a streptavidin-R-phycoerythrin conjugate (the reporter fluorophore), enriching beads with the reporter in proportion to ligase joining. Finally, each bead is identified (indicating what SNP is being detected) and evaluated for reporter fluorophore enrichment (indicating how much of that SNP was present) in a liquid flow as they pass through the optical chamber in the flow fluorimeter.

Our efforts to detect minority variant LDR products on a suspension array revealed significant signal and background fluctuations which prevented accurate calculation of sample SNP content, as shown previously [9], [10]. In this report, we have characterized two discrete hybridization artifacts which cause signal fluctuations and thereby confound LDR detection on an array. Specifically: 1) carryover template DNA elicits false signal by tethering the RCO to the bead-bound BCO in the absence of ligation and 2) unpredictable BCO secondary structures interfere with bead binding, suppressing *bona-fide* array signal. These observations informed design of competimer and duplex nucleator oligonucleotides to induce benign DNA hybrids, disrupting the unwanted structures and significantly improving both signal and background. Used together, duplex nucleator and competimer oligonucleotides improved detection sensitivity by more than an order of magnitude and, in some cases, enabled quantification of previously undetectable sequences, as demonstrated by a model assay for HIV-1 drug resistance mutations.

## Results

### A Multiplex Assay for Drug Resistant Mutations in HIV-1 Pol

**Figure 2:**
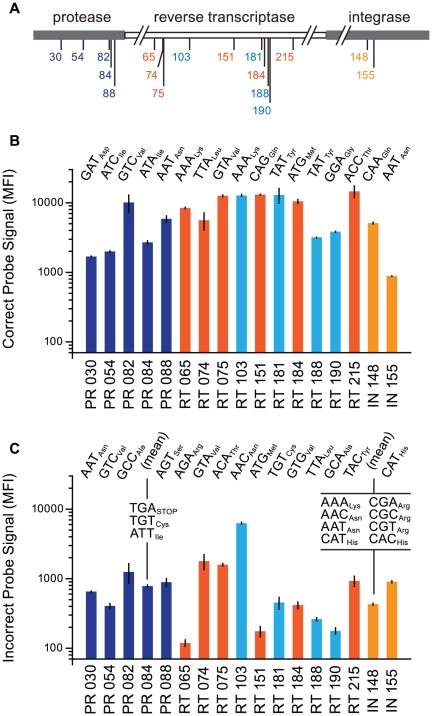
Prototype assay for 17 drug resistance mutations in HIV-1 Pol. (A) Schematic representation of HIV-1 *pol* gene and relative locations of drug resistance-associated codons in HIV-1 protease (PR), reverse transcriptase (RT) and integrase (IN) coding regions. (B) Correct signal from the prototype assay are shown with amplitude in units of median fluorescence intensity (MFI; calculated for ≥75 beads per well; +/−s.d., N = 3 independent experiments). (C) Incorrect signal amplitude in the prototype assay (+/−s.d., N = 3 independent experiments). *Multiple incorrect signals were averaged for PR-084 and IN-148 detections.

Five classes of antiretroviral drugs, i.e. protease inhibitors (PIs), nucleoside analogue reverse transcriptase inhibitors (NRTIs), non-nucleoside reverse transcriptase inhibitors (NNRTIs), integrase inhibitors (INIs) and entry inhibitors, are commonly prescribed as part of highly active antiretroviral treatment regimen, but discrete mutations conferring resistance are observed for only the PI, NRTI, NNRTI, and INI drug classes. For this study, seventeen primary HIV-1 drug resistance-associated mutations were chosen based on definitive phenotypic resistance profiles in HIV-1 subtype B infections [11] (hivdb.stanford.edu, accessed in April 2008; Figure 2A). In oligonucleotide probe design, each BCO was amended with a 5′-terminal “tag”, an HIV-unrelated 24-nt DNA sequence which is complementary to one of the bead-coupled oligonucleotides (anti-tags) available from the bead manufacturer (LUA-#; Table S1).

Initial assay performance was demonstrated using a wild-type amplicon, including most of the HIV-1 pol gene, comprising protease (PR), reverse transcriptase (RT) and integrase (IN) coding sequences, as the template DNA for ligase discrimination. As summarized in Figure 2, each drug resistance site was probed with an RCO-BCO set (typically one BCO for the mutant sequence, one BCO for the wild-type sequence and an RCO which can be ligated to either the mutant or wild-type BCO). The template DNA was diluted in series, combined with all assay probes (35 BCOs and 20 RCOs) and subjected to ligase discrimination. After LDRs were complete, suspension array beads (one region for each of the 35 BCOs), and then a fluorescent reporter (Streptavidin – R-phycoerythrin), are added to all wells. Subsequent flow fluorimetry assesses reporter enrichment for each bead region and, since the reporter fluorophore should only associate with beads via RCO linkage to the BCO, reporter associated with a specific bead region reflects how much of the region-associated SNP was present in the template DNA. The multiplex OLA gave high median fluorescent intensity (MFI >10,000) for the majority of sites except for RCO-BCO_wtIN-151_ (<1000 MFI) and BCO-RCO_wt_ combinations for protease codons 30, 54, and 84 (<3000 MFI; Figure 2B).

Incorrect probe signal amplitudes were more varied (Figure 2C). RCO-BCO combinations for RT codons 65, 151, 188 and 190 had the lowest incorrect signals (less than 300 MFI compared to a mean incorrect signal of 1031 MFI) whereas those for PR codon 82 and RT codons 74, 75 and 103 exhibited the highest incorrect signals in the dataset (>1000 MFI). These background signals were unexpected based on previous results with a radioactive protocol yielding significantly lower background [12].

### Characterization of Template-Tethering

**Figure 3:**
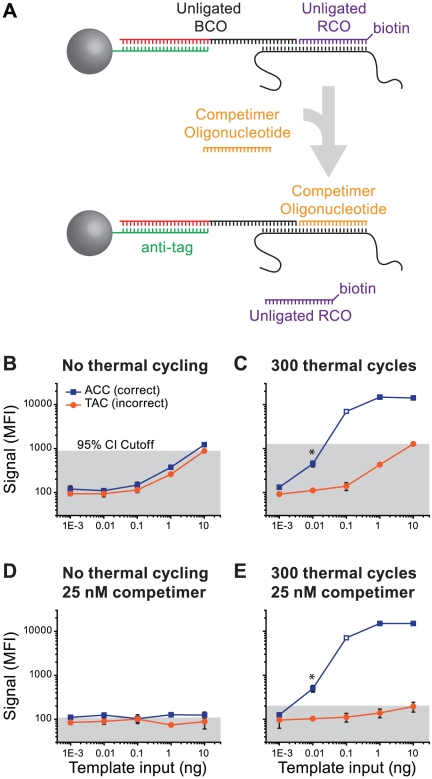
Template tethering. (A) Illustration of competimer oligonucleotide function. Unligated RCOs are tethered to an unligated BCO via the template DNA during bead hybridization (top). A competimer oligonucleotide displaces the RCO from the bead-bound species. (B–E) Demonstration of template tethering and effects of a competimer oligonucleotide (all +/−s.d., N = 4 independent experiments). Template DNA (2 ng/µl) was diluted with λ-phage DNA at the same concentration. Ligase discrimination reactions were assembled and subjected to 300 thermal cycles or no thermal cycling (mock). Grey fill indicates the 95% CI calculated from the incorrect signal. (B) Mock series demonstrates false signal in the absence of ligase activity. (C) Reactions subjected to thermal cycling demonstrates SNP discrimination (blue) and incorrect signal similar to panel B (orange). (D) Mock series subjected to bead hybridization with 25 nM competimer demonstrates false signal suppression. (E) Reactions subjected to thermal cycling and bead hybridization with 25 nM competimer lack the false signal (orange) shown in panel C. Data for 0.1 ng template input were not significantly different than the same data point in panel C (open blue square in both panels; MWW p = 0.47).

To rule out ligase error as the source of high incorrect signal, we used an 839 bp amplicon (RTS2-RTA8) from HIV-1 reference plasmid pNL4.3 which includes the wild-type reverse transcriptase codon 215 ACC. Ligase discrimination reactions and a mock reaction series (no thermal cycling) were assembled with increasing amounts of template DNA (0.01 to 10 nanograms per reaction) with wild-type RCO+BCO_wt RT-Thr215 ACC_ and mutant RCO+BCO_mut RT-Tyr215 TAC_ probes (Figure 3B–E). As expected, the correct signal rose sharply in proportion to template input with thermal cycling (Figure 3C, blue trace). However, the incorrect signal increased to the same extent with or without thermal cycling (Figure 3B–C, orange trace). Ultimately, nearly 1000 MFI of incorrect signal was generated by 10 ng template DNA with and without enzymatic activity, indicating that the majority of non-specific signal was ligase-independent, i.e. not caused by ligase error.

### Competimer Oligonucleotides Inhibit Template Tethering

Carryover template DNA might interact with the RCO and/or BCO during bead hybridization. If the template DNA interacts with both the BCO and RCO, it could provide a link between unligated RCOs and the suspension array beads, independent of ligation (Figure 3A, top). To test this hypothesis, a sense-stranded oligonucleotide was designed with the same sequence as the RCO, but lacking both a 5′-phosphate and 3′-biotin (“competimer”; Table S3; Figure 3A, bottom). The competimer oligonucleotide eliminated template-dependent incorrect signal from all reactions regardless of cycling conditions (Figure 3D–E), consistent with a model in which template DNA can tether the RCO to bead-bound BCOs in the absence of ligation (Figure 3A). Furthermore, the correct signal from a 0.01 ng control input was insignificant without competimer due to high incorrect signal (Figure 3C) whereas competimer supplementation extricated this data point (p = 0.03; Mann-Whitney; Figure 3E). Thus, formation of an alternative hybrid between the competimer and the template DNA during bead hybridization displaced unligated RCOs from the bead-bound species and resulted in an estimated 10-fold increased sensitivity.

### Predicted BCO Secondary Structures

We next examined the disparity among correct signals from the prototype assay. A subset of BCOs with a range of reactivities (Figure 2B) were analyzed for secondary structure (Figure S2) using a standard algorithm [13]. Results implicated secondary structure as a possible cause of low signal with BCO, but we suspected that predictions were inaccurate since they did not account for TMAC used in hybridizations (2.5 M final) or deoxyinosine and deoxyuracil substitutions in our BCOs, so BCO-bead binding affinities were measured *in-vitro*.

**Figure 4:**
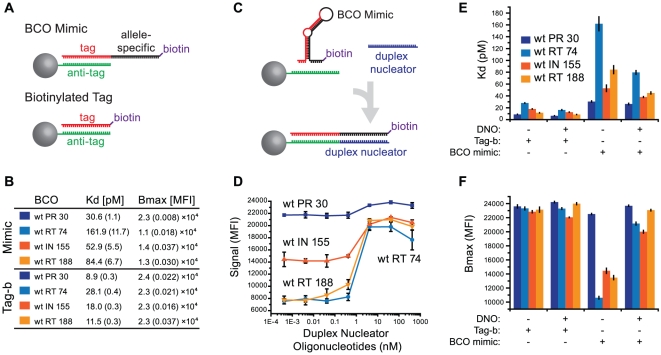
BCO mimic and tag binding parameters. (A) Illustration of reagents used for the BCO binding assay. Bead-conjugated antitag sequences (green) bind tag sequences (black) during normal bead hybridization. BCO mimics (top) are identical to the corresponding BCO (allele-specific sequence in red), but have 3′-biotin modifications to elicit suspension array signal without the need for ligation. Biotinylated tag sequences (tag-b; bottom) are simply BCO mimics lacking the allele-specific sequence. (B) BCO mimic (top) and tag-b (bottom) dissociation constant (K_d_) and maximal binding capacity (B_max_) values (+/−s.d. in parentheses, N = 4 independent experiments). (C) Secondary structures in BCO mimics (top) preclude tag-antitag binding. Duplex nucleator oligonucleotides (DNO; blue; bottom) form a duplex with the BCO allele-specific region, liberating the tag sequence to bind the bead-bound antitag. (D) DNOs were titrated into bead hybridizations containing 0.42 nM each BCO mimic or biotinylated oligonucleotide and detected by flow fluorimetry. (E and F) The experiment in panel B was repeated with and without 2.5 nM DNOs to determine their effects on BCO mimic binding binding as measured by K_d_ (E) and B_max_ (F) values (all +/−s.d., N = 4 independent experiments).

BCO sequences corresponding to BCO_wtPR 30_, BCO_wtRT 74_, BCO_wtIN 155_ and BCO_wtRT 188_ were modified with a 3′-biotin (BCO mimics; Figure 4A, top) to elicit suspension array signal without prior LDR and allow functional assessment of bead binding. Binding parameters for each BCO mimic was compared to that of its 3′-biotinylated tag (i.e. a BCO mimic lacking the allele-specific sequence; Figure 4A, bottom). Since biotinylated tag/BCO mimic pairs differ only by the presence of the allele-specific sequence (present in the BCO mimic, absent in the biotinylated tag; Figure 4A), differential binding can be directly attributed to the BCO allele-specific sequence.

BCO mimic bead dissociations constants (K_d_) and maximal binding capacities (B_max_) were compared to those of their biotinylated tag sequences (Figure 4B, “tag-b”), wherein the BCO_wt RT 74_ mimic served as a reference for high-level correct signal in the prototype assay and BCO_wt PR 30_, BCO_wt RT 188_, and BCO_wt IN 155_ as references for weak signals (Figure 2B). Interestingly, BCO_wt RT 74_ mimic bead binding was the least efficient in the BCO mimic subset. In all cases, tag-b sequences bound beads more efficiently (>3-fold) than BCO mimics containing an identical sequence (K_d_ = 162 pM for BCO_wt RT 74_ mimic versus 8.9 pM for the comparable tag-b). Binding maxima followed a similar trend: that for the BCO_wt PR 30_ mimic was similar to its tag-b sequence and other BCO mimics were impaired (Figure 4B). BCO mimic binding profiles partially explained BCO reactivity in the prototype assay, but close correlation was not expected since BCO mimic signal is independent of LDR probe reactivity. Regardless, these data demonstrate that BCO allele-specific sequences interfere with tag-antitag recognition, indicating minor variant detection with the multiplex OLA was hampered by impaired BCO binding and assay variability was due in part to variability in BCO binding (BCO mimic B_max_ coefficient of variation  = 0.33 for the data set).

### Duplex Nucleators Improve BCO Mimic Binding

To mitigate the negative effects of BCO secondary structures on bead binding, duplex nucleator oligonucleotides were designed (DNOs; Table S4; Figure 4C) to complement the allele-specific sequence of each BCO. In each case, inclusion of DNOs in the bead hybridization step increased signal from BCO mimics, ranging from a slight (Figure 4D; BCO_wt RT 30_ mimic) to near 3-fold (BCO_wt RT 188_ mimic).

BCO mimic binding was assessed in the presence of 25 nM each DNO (Figure 4E & F). DNOs decreased BCO mimic K_d_ values from 1.2-fold (Figure 4E; BCO_wt RT 30_ mimic), to 2-fold (BCO_wt RT 74_ mimic). Of greater consequence, DNOs increased B_max_ values for all BCO mimics to greater than 21,000 MFI (Figure 4F). For example, BCO_wt RT 74_ mimic had the lowest B_max_ of all tested, but DNO supplementation increased this value by 2-fold, normalizing to DNO-supplemented values for other BCO mimics (overall CV = 0.074). Overall, we observed that DNO supplementation improved BCO binding, consistent with a model in which efficient tag/anti-tag recognition is precluded by secondary structure in the BCO (Figure 4C), as well as with recent studies demonstrating oligonucleotide binding cooperativity between two oligonucleotides binding to adjacent sites on a single-stranded DNA [14].

### Improved Prototype OLA Performance

In light of improved BCO-bead binding with DNOs, multiplex OLA performance was evaluated using DNOs in conjunction with competimers. Duplex nucleator and competimer oligonucleotides were designed to anneal to the allele-specific portion of each BCO (compare Figure S1 to Figure 4C) for all remaining sites in the prototype assay (Table S3 & S4). Rigorous assay characterization also required a standard control template. Since the HIV-1 subtype B pol consensus sequence differs from those of common laboratory adapted strains, we adapted an oligonucleotide assembly procedure [15] (Figure S3) to produce clinically-relevant templates (Table S7). Control sequences, comprising all BCO and RCO binding sequences in the consensus subtype B HIV-1 pol, were constructed by ligating 10 short (<100 nt) oligonucleotides (Table S6) with degenerate positions corresponding to the mutations of interest (Figure 3A). The single-stranded ligation product was then PCR amplified and cloned to produce six variants, differing only in nucleotides corresponding to drug resistance-associated codons (Table S7). Because drug-resistant HIV-1 variants often exist as minor components of an otherwise drug-susceptible HIV-1 patient infection, PCR amplicons from these constructs were paired and mixed in different proportions (a “solute” template was diluted by a “solvent” template) for analysis by multiplex OLA.

**Figure 5:**
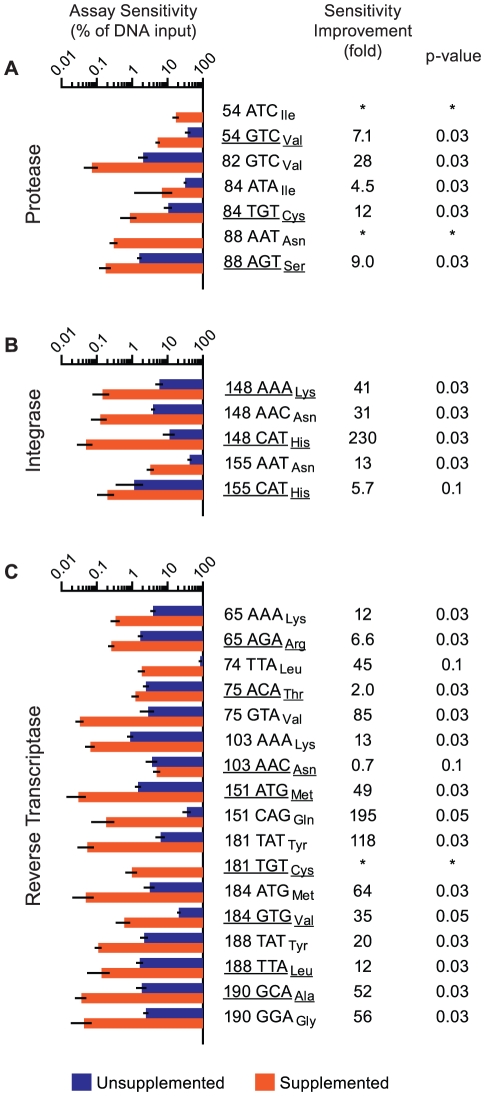
Duplex nucleator and competimer oligonucleotides improve assay sensitivity. Abridged consensus subtype B HIV-1 Pol sequences containing all OLA probe binding sites were produced by ligase-mediated oligonucleotide assembly (Materials and Methods; Figure S3). Amplicons from these constructs differed only in the nucleotides corresponding to the drug resistance-associated codons. Each synthetic amplicon (solute) was diluted in series with another at the same concentration (solvent), used as templates in an ligase discrimination reaction and then subjected to bead hybridization (unsupplemented) or that with 25 nM competimer and 2.5 nM duplex nucleator oligonucleotides (supplemented). Assay sensitivity values reflect the minimal solute template content (%) required to elicit the cutoff value for the unsupplemented (blue bars) and supplemented assay (orange bars) (all +/−s.d., N = 4 independent experiments). Codons associated with drug resistance are underlined. Fold sensitivity improvement values >1 reflect detections where oligonucleotide supplementation gave a lower sensitivity value (middle column). Mann-Whitney Rank Sum Test p-values are shown at the right. Assay sensitivity for (A) seven codon sequences in PR, (B) five codon sequences in IN and (C) seventeen codon sequences in RT. *Sensitivity improvements could not be determined for codons PR 54 ATC_Ile_, PR 88 AAT_Asn_ and RT 181 TGT_Cys_ because these codons were undetectable in the unsupplemented assay.

LDRs were detected in the presence or absence of 320 nM each competimer and 25 nM each duplex nucleator oligonucleotide (both added in bead hybridization) and analyzed by flow fluorimetry (Figure S4). Assay sensitivity values for those codons unique to the solute template were determined by fitting 4-parameter logistic curves to the corresponding assay signal and calculating the solute content required to elicit the cutoff value (95% CI calculated from the solvent template alone; Figure 5).

In the absence of competimer and duplex nucleator oligonucleotides (unsupplemented), the log-average sensitivity of this multiplex OLA system was 5.1% SNP content. Assay sensitivity was significantly improved (Mann-Whitney p≤0.05) for 21 of 29 BCO+RCO combinations when the competimer and DNO were included (supplemented) in the bead hybridization step (log-average sensitivity  = 0.24% SNP content; overall sensitivity improvement  = 21-fold). The greatest improvements were observed in detecting codons IN 148 CAT_His_ (Figure 5B), RT 151 CAG_Gly_, RT 181 TAT_Tyr_, RT 75 GTA_Val_, RT 184 ATG_Met_, RT 190 GCA_Ala_ and RT 190 GGA_Gly_ (Figure 5C; all >50-fold). The poorest sensitivity values were observed for codons associated with PR inhibitor resistance (Figure 5A), despite significant improvements for five (all p = 0.03, 4.5 to 28 fold). Sensitivity values for other RT codons (Figure 5C) were significantly improved (p≤0.05, 2.0 to 195 fold) for less than 0.5% SNP content, with the exception of 74 TTA_Leu_ and 103 AAC_Asn_. Although assay sensitivity for RT 74 TTA_Leu_ was improved 45-fold, significance for improvement could not be established (p = 0.1) since the unsupplemented assay signal fitted curves failed validation in two of four experiments (Figure S5). Assay sensitivity for RT 103 AAC_Asn_ was unique in the dataset in that it was the only codon detection not improved by oligonucleotide supplementation (p = 0.1). Interestingly, wild-type RT 103 AAA_Lys_ was the only codon detectable at less than 1% in a mixture without supplementation (Figure 5C; Table 1).

**Table 1:** Assay performance is improved by oligonucleotide supplementation.

Sensitivity Bin*	Number of Codons	
	Unsupplemented	Supplemented
**Undetectable**	3	0
**10<S≤100**	8	1
**1<S≤10**	17	6
**0.1<S≤1**	1	13
**0.01<S≤0.1**	0	9

*lowest detectable content

## Discussion

A multiplex OLA for 17 drug resistance-associated mutations in HIV-1 Pol demonstrated unexpectedly high levels of incorrect signal on a Luminex suspension array, in contrast to previous studies showing minimal ligase error when ligation products were analyzed by denaturing polyacrylamide gel electrophoresis [12], [16]. To reconcile this inconsistency, we investigated the cause of high incorrect signal and low correct signal for polymorphism detection using multiplex OLA. Mock reactions analyzed by flow fluorimetry provided evidence that high incorrect signal was not ligase-dependent. We proposed that template-dependent incorrect signals were the result of discrete artifacts created during bead hybridization wherein template DNA carried over from the ligation reaction tethered unligated RCOs to unligated BCOs on the bead. This model was supported by the observation that this ligase-independent signal was silenced by an oligonucleotide (competimer) designed to displace the unligated RCO, increasing assay sensitivity approximately 10-fold.

Oligonucleotide folding thermodynamic calculations indicated that secondary structures in specific BCO sequences partially obscured tag residues, so we examined BCO binding quantitatively. Binding experiments demonstrated that BCO allele-specific sequences impaired tag-antitag interaction to a greater extent than predicted by a standard algorithm for secondary structure. BCO binding was then rescued by inducing duplex formation in the BCO allele-specific sequences with an additional oligonucleotide (duplex nucleator oligonucleotide; DNO), supporting our model for non-tag sequence interference and, more importantly, providing an invaluable reagent for the multiplex assay.

Multiplex OLA sensitivity was characterized using template DNA sequences consisting of an abridged subtype B HIV-1 Pol gene consensus and six different permutations of drug resistance mutations. Each amplicon was diluted in series with another at the same concentration and the resulting mixture was used as a template for multiplex OLA. Assays supplemented with duplex nucleator and competimer oligonucleotides gave higher correct signals and lower incorrect signals than our base protocol, yielding an average 21-fold gain in assay sensitivity. Moreover, oligonucleotide supplementation produced high signal for three polymorphisms which were previously undetectable (Table 1, Figure S5). As a result, 15 wild type and 14 mutant codons could be accurately and simultaneously detected at a frequency as low as 0.03% (average 0.24%).

Fundamental parallels between suspension arrays and solid-state microarrays suggest a wider application for simplifying unintended nucleic acid target structures. For example, the Noble group reported that rigorous thermodynamic calculations could not account for variations among solid-state microarray probe signals corresponding to the same rRNA target molecule [17]. In our system, we improved target binding (the BCO mimic is the target in this case) to an immobilized probe (the antitag) by occupying an adjacent site on the target with a third oligonucleotide. It might then be possible to normalize solid-state RNA (and DNA) microarray signals for the same target by inducing duplex formation in target sequences adjacent to the probe binding site using a similar strategy. Moreover, DNOs and competimers may also improve and expand the utility of “next-generation” array systems using holographic bar-coded (Illumina), fluorescence-encoded (Parallume, Luminex) or high-efficiency scattering (Nanosphere) particles in suspension.

The current limits of array-based SNP analysis was demonstrated by a recent study using a similar method (without artifact silencing) and rigorous background modeling to account for template-dependent background [10]. After data transformation, the authors found that a minority variant must comprise 10% of the sample to attain a call rate of 67%. Thus, the improvements described herein provide 40-fold greater sensitivity (0.25% mutant content) than mathematical deconvolution and allow this assay platform to be used for entirely new methodologies, such as those for detection of acquired oncogenic polymorphisms and RNA splice variants as well as pathogen mutations associated with drug resistance or adverse clinical outcomes. As they relate to our model system, several studies now suggest that initial presence of minority drug-resistant HIV-1 variants can predict their eventual dominance and subsequent ARV drug failure [18], [19]. Minority drug-resistant HIV-1 variants may also act as latent reservoirs for therapeutic inefficacy, since they often persist in patients after treatment interruption or ARV drug regimen change [19], [20]. Thus, early detection of low frequency drug resistance mutations using multiplex OLA may prolong drug utility and deter HIV-1 disease progression. Toward this end, 17 of the most common HIV-1 mutations (associated with resistance to NNRTIs, NRTIs, PIs, and INIs) may be rapidly and simultaneously detected at frequencies indicative of unfavorable treatment and disease outcomes. More broadly, the rationale we used to characterize and resolve suspension array artifacts may provide a means for others to obtain more accurate and higher quality information from nucleic acid arrays.

## Materials and Methods

### Oligonucleotide Design

One hundred seventy-four subtype B HIV-1 Pol coding sequences were obtained from Los Alamos HIV database (www.hiv.lanl.gov) in April 2008. Oligonucleotide probes were designed to anneal at 75°C under LDR conditions (40 mM Na^+^ and 7.5 mM Mg^2+^) and 5′-amended with one of 100 tag sequences (Luminex Corp, Austin, TX). Reference alignment polymorphisms occurring with ≥10% frequency were accommodated with non-standard bases (deoxyuridine to accommodate frequent purine transitions or transversions in the negative strand; deoxyinosine to accommodate frequent pyrimidine transitions) in the probe sequences, as previously reported [16]. Probe codon specificities corresponded to mutations most frequently observed in the Stanford HIV Drug Resistance Database (hivdb.stanford.edu, accessed April 2008; Figure 2A). Secondary structure thermodynamics were predicted using the DINA Melt Two-State Folding interface [13] in 10 mM Na^+^ and 1.5 mM Mg^2+^. BCOs are shown in Table S1, RCOs in Table S2, competimer oligonucleotides in Table S3, and duplex nucleator oligonucleotides (DNOs) in Table S4.

### Positive Control Templates

Plasmid pNL4.3 was PCR-amplified with either ML-gag-F3 (nt 2047-2081, HXB2 numbering) and ML-int-R2 (nt 4763–4799), or RTS2 (nt 2691–2715) and RTA8 (nt 3505–3529; Table S5) with Platinum Taq DNA Polymerase (Invitrogen) at 95°C (2 min.), 35 cycles of 95°C (30 sec.), 55°C (30 sec.), 72°C (1 min.) and final extension at 72°C (10 min.). Amplicons were purified using the Qiagen PCR Cleanup Kit, verified by agarose gel electrophoresis, quantified using the PicoGreen fluorescence assay (Invitrogen) on a Perkin-Elmer Victor 3 fluorescent plate reader (Perkin-Elmer; Waltham, MA) and diluted to 2 ng/µl in ultrapure water and then, for Figures 2 and 3, with λ-phage DNA (2 ng/µl).

### Control Templates Produced by Ligase-Mediated Oligonucleotide Assembly

Control DNA sequences for Figures 5 and S4 were produced similar to that reported previously [15] (details of our adaptation are shown in Figure S3). The HIV-1 subtype B consensus sequence was trimmed to OLA probe target regions and divided into 10 shorter (<100 nt) segments to produce 5′-phosphorylated (assembly) oligonucleotide sequences. Nine additional (junction span) oligonucleotides were designed to define the order of assembly oligonucleotide joining (Figure S3A). The resulting 19 oligonucleotides (AP-01 through AP-10 and JS-01 through JS-09; Table S6) were combined (2.5 nM each) with 5 Units Ampligase DNA ligase and 1X Ampligase buffer in 50 µl, heated to 95°C and slowly cooled to 20°C. Five microliters of the assembly reaction was PCR-amplified using primers LigPol-F2 and LigPol-R2 (200 nM final, Table S5, Figure S3B) with Platinum Taq DNA Polymerase and 1X Platinum Taq buffer in 50 µl using the PCR conditions above. PCR products were cloned into pCR2.1-TOPO and grown in TOP10 cells (Invitrogen). DNA sequences were verified by DNA sequencing (ACGT, Inc.). Plasmid inserts were PCR amplified (LigPol-F2 and LigPol-R2; same conditions as above, Figure S3C), processed as above and diluted to 5 ng/µl.

### Ligase Discrimination Reactions (LDRs)

Except where indicated otherwise, ligase discrimination reactions used 10 ng (Figure 2) or 25 ng (Figures 5 and S4) template DNA, 5 units of Ampligase DNA ligase (Epicentre Biotechnologies, Madison, WI) in a 12 µl reaction containing 7.5 nM each oligonucleotide probe, 15 mM Tris-HCl pH 8.3, 0.06% Triton-X100, 1 mM dithiothreitol, 40 mM KCl, 7.5 mM MgCl_2_, 0.3 mM nicotinamide adenine dinucleotide sodium salt and 0.08% polyethylene glycol (mean molecular weight ∼6000 Da) and subjected to 150 or 300 cycles (where indicated) of 95°C (10 sec.) and 37°C (40 sec.).

### Bead and Streptavidin-R-Phycoerythrin Hybridization, Flow Fluorimetry

Approximately 750 of each xTAG bead, competimer and/or DNO (where indicated) were added to each LDR in 1X TMAC buffer (2.5 M tetramethylammonium chloride (TMAC), 0.1 M Tris-HCl (pH 8.0), 3 mM ethylenediaminetetraacetic acid and 0.1% sodium dodecyl sulfate) in 60 µl and subjected to 95°C (5 min.) and 37°C (45 min.), followed by addition of a Streptavidin-R-phycoerythrin conjugate (100 ng/well; Invitrogen #S-866) in 6 µl 1X TMAC buffer and incubation at 37°C (45 min.). Seventy-five beads per region were measured on a Luminex 200 instrument with BioPlex Manager software (Bio-Rad Life Sciences, Hercules, CA) at the highest reporter channel photomultiplier tube voltage setting (typically 700–750 V).

### Sensitivity Estimation, Curve Fitting and Statistical Analyses

BCO mimic and tag-b K_d_ and B_max_ values were calculated in Origin 8 (OriginLab Corp, Northampton, MA) using a 4-parameter logistic equation. For assay sensitivity experiments, cutoff values were determined by calculating the 95% CI for the signal elicited by the solvent template alone. Correct signal amplitude was modeled in Origin 8 as a function of the solute HIV-1 amplicon (given as % of total input) and by curve fitting (as above). Curve fitting parameters were used to determine the minimal template DNA input required to elicit the cutoff value. Statistical analyses were performed using the Mann-Whitney-Wilcoxon Rank Sum Test implementation in Minitab 15 (State College, PA) wherein p≤0.05 was considered significant.

## Supporting Information

Figure S1 **Duplex nucleator oligonucleotides facilitate bead binding.** For analysis of LDR products, duplex nucleator oligonucleotides bind to the allele-specific region of the ligated BCO, similar to that shown in Figure 4C. (EPS)

Figure S2 **Predicted BCO secondary structures.** BCO sequences were folded using the DINAMelt Server interface (http://dinamelt.bioinfo.rpi.edu/) in 10 mM Na^+^ and 1.5 mM Mg^2+^, corresponding to bead hybridization conditions. Red outlines indicate tag sequences. (A) BCO_wt PR 30_ was absent of predicted secondary structure in the tag region. (B) BCO_wt RT 74_ was predicted to form a weak hairpin at the 3′-end but no secondary structure in the tag sequence. (C) BCO_wt RT 188_ formed a complex secondary structure at the 3′-end of the sequence. (D) BCO_wt IN 155_ predicted secondary structure involved 8 nt of the tag sequence. (EPS)

Figure S3 **Ligase-mediated oligonucleotide assembly.** The HIV-1 subtype B consensus sequence was constructed from 10 5′-phosphorylated “assembly” oligonucleotides (AP#) assembled in an order defined by nine 3′-aminated “junction span” oligonucleotides (JS#). Twenty-four degenerate positions corresponding to codons of interest are depicted by red circles (some mutant codons differed from the wild-type sequence by two or three nucleotides). (A) Assembly and junction span oligonucleotides are combined in a single tube with a thermostable template-dependent DNA ligase. Magnification shows 5′-phosphate and 3′-amine modifications on the assembly and junction span oligonucleotides, respectively. The reaction is heated and slowly cooled to produce a single-stranded DNA with the desired sequence. (B) The single stranded ligation product is PCR amplified from the 5′- and 3′-ends to produce an amplicon with the desired sequence and heterogeneity in codons of interest. (C) The bulk amplicon was cloned (see Materials and Methods) and verified by DNA sequencing. Six variants were selected for PCR amplification and for further analysis by multiplex OLA (Table S7). (EPS)

Figure S4 **Oligonucleotide supplementation increases correct signal, decreases incorrect signal.** Abridged consensus subtype B HIV-1 Pol sequences containing all OLA probe binding sites were produced by ligase-mediated oligonucleotide assembly (Figure S3), cloned and PCR-amplified to produce amplicons which differed only in the nucleotides corresponding to the drug resistance-associated codons. Synthetic amplicons (solute) were diluted in series with another at the same concentration (solvent), used as LDR templates and subjected to either normal bead hybridization (unsupplemented) or that with 25 nM competimer and 2.5 nM duplex nucleator oligonucleotides (supplemented). Signals from the correct (“100% solute”) and incorrect (“100% solvent”) synthetic templates corresponding to (A) seven codon sequences in PR, (B) five in IN and (C) seventeen in RT (all +/−s.d., N = 4). (EPS)

Figure S5 **RT-74-TTA_Leu_ did not produce evaluable trends in two of four experiments without oligonucleotide supplementation.** Data from two unsupplemented experiments (grey traces) detecting RT-74-TTA_Leu_ failed to direct the curve-fitting routine to convergence. Orange traces depict unsupplemented experiments for which curve fitting converged. Blue traces depict signals from four experiments with oligonucleotide supplementation. (EPS)

Table S1 **Bead capture oligonucleotides.** (DOC)

Table S2 **Reporter capture oligonucleotides.** “p-” and “-b” depict 5′-phosphate and 3′-biotin modifications, respectively. (DOC)

Table S3 **Competimer oligonucleotides.** (DOC)

Table S4 **Duplex nucleator oligonucleotides.** (DOC)

Table S5 **PCR Oligonucleotides.** (DOC)

Table S6 **Assembly and junction span oligonucleotides for ligase-mediated oligonucleotide assembly.** “p-” and “-amine” depict 5′-phosphate and 3′-amine modifications, respectively. (DOC)

Table S7 **Synthetic HIV-1 subtype B consensus clone sequences.** (DOC)

## References

[1] Dent AE, Yohn CT, Zimmerman PA, Vulule J, Kazura JW (2007). A polymerase chain reaction/ligase detection reaction fluorescent microsphere assay to determine Plasmodium falciparum MSP-119 haplotypes.. Am J Trop Med Hyg.

[2] McNamara DT, Kasehagen LJ, Grimberg BT, Cole-Tobian J, Collins WE (2006). Diagnosing infection levels of four human malaria parasite species by a polymerase chain reaction/ligase detection reaction fluorescent microsphere-based assay.. Am J Trop Med Hyg.

[3] Einstein MH, Leanza S, Chiu LG, Schlecht NF, Goldberg GL (2009). Genetic variants in TAP are associated with high-grade cervical neoplasia.. Clin Cancer Res.

[4] Li J, Wang L, Mamon H, Kulke MH, Berbeco R (2008). Replacing PCR with COLD-PCR enriches variant DNA sequences and redefines the sensitivity of genetic testing.. Nat Med.

[5] Cai F, Chen H, Hicks CB, Bartlett JA, Zhu J (2007). Detection of minor drug-resistant populations by parallel allele-specific sequencing.. Nat Methods.

[6] Taylor JD, Briley D, Nguyen Q, Long K, Iannone MA (2001). Flow cytometric platform for high-throughput single nucleotide polymorphism analysis.. Biotechniques.

[7] Carson RT, Vignali DA (1999). Simultaneous quantitation of 15 cytokines using a multiplexed flow cytometric assay.. J Immunol Methods.

[8] Villahermosa ML, Beck I, Perez-Alvarez L, Contreras G, Frenkel LM (2001). Detection and quantification of multiple drug resistance mutations in HIV-1 reverse transcriptase by an oligonucleotide ligation assay.. J Hum Virol.

[9] Carnevale EP, Kouri D, DaRe JT, McNamara DT, Mueller I (2007). A multiplex ligase detection reaction-fluorescent microsphere assay for simultaneous detection of single nucleotide polymorphisms associated with Plasmodium falciparum drug resistance.. J Clin Microbiol.

[10] DaRe JT, Kouri DP, Zimmerman PA, Thomas PJ (2010). Differentiating Plasmodium falciparum alleles by transforming Cartesian X,Y data to polar coordinates.. BMC Genet.

[11] Cooper DA, Steigbigel RT, Gatell JM, Rockstroh JK, Katlama C (2008). Subgroup and resistance analyses of raltegravir for resistant HIV-1 infection.. N Engl J Med.

[12] Troyer RM, Lalonde MS, Fraundorf E, Demers KR, Kyeyune F (2008). A radiolabeled oligonucleotide ligation assay demonstrates the high frequency of nevirapine resistance mutations in HIV type 1 quasispecies of NVP-treated and untreated mother-infant pairs from Uganda.. AIDS Res Hum Retroviruses.

[13] Markham NR, Zuker M (2008). UNAFold: software for nucleic acid folding and hybridization.. Methods Mol Biol.

[14] Rahimian M, Miao Y, Wilson WD (2008). Influence of DNA structure on adjacent site cooperative binding.. J Phys Chem B.

[15] Chen HB, Weng JM, Jiang K, Bao JS (1990). A new method for the synthesis of a structural gene.. Nucleic Acids Res.

[16] Lalonde MS, Troyer RM, Syed AR, Bulime S, Demers K (2007). Sensitive oligonucleotide ligation assay for low-level detection of nevirapine resistance mutations in human immunodeficiency virus type 1 quasispecies.. J Clin Microbiol.

[17] Pozhitkov AE, Tautz D, Noble PA (2007). Oligonucleotide microarrays: widely applied—poorly understood.. Brief Funct Genomic Proteomic.

[18] Descamps D, Chazallon C, Loveday C, Bacheler L, Goodall R (2009). Resistance and virological response analyses in a three initial treatment strategy trial: a substudy of the INITIO trial.. HIV Clin Trials.

[19] Varghese V, Shahriar R, Rhee SY, Liu T, Simen BB (2009). Minority variants associated with transmitted and acquired HIV-1 nonnucleoside reverse transcriptase inhibitor resistance: implications for the use of second-generation nonnucleoside reverse transcriptase inhibitors.. J Acquir Immune Defic Syndr.

[20] Palmer S, Boltz V, Martinson N, Maldarelli F, Gray G (2006). Persistence of nevirapine-resistant HIV-1 in women after single-dose nevirapine therapy for prevention of maternal-to-fetal HIV-1 transmission.. Proc Natl Acad Sci U S A.

