# Supplementary material for: DNA Suspension Arrays: Silencing Discrete Artifacts for High-Sensitivity Applications
Source: PLoS One. 2010 Nov 8;5(11):e15476. doi: 10.1371/journal.pone.0015476 (PMC2975679; doi:10.1371/journal.pone.0015476)
Supplement: Table S1 — Bead capture oligonucleotides. (DOC) [file pone.0015476.s006.doc]

**Table S1**: Bead Capture Oligonucleotides (BCOs)

| **Name** | **Sequence** | **Region** | **Bead Association** |
| --- | --- | --- | --- |
| **STBIN-148-AAX** | TTCAATCATTCAAATCTCAACTTTGGCATTCCCTACAATCCCCAAAGTAA | IN | LUA-023 |
| **STBIN-148-CAX** | CTTTTACAATACTTCAATACAATCGGCATTCCCTACAATCCCCAAAGTCA | IN | LUA-020 |
| **STBIN-148-CGX** | AATCCTTTCTTTAATCTCAAATCAGGCATTCCCTACAATCCCCAAAGTCG | IN | LUA-021 |
| **STBIN-155-AAX** | CTTTAATCCTTTATCACTTTATCAATTCCCTACAATCCCCAAAGTCAAGGAGTAITAGAATCTATGAA | IN | LUA-017 |
| **STBIN-155-CAX** | TCAAAATCTCAAATACTCAAATCAATTCCCTACAATCCCCAAAGTCAAGGAGTAITAGAATCTATGCA | IN | LUA-018 |
| **STBPR-030-AAX** | TACACTTTATCAAATCTTACAATCGGGCAICTAAAGGAAGCTCTATTAGATACAGGAGCAGATAA | PR | LUA-003 |
| **STBPR-030-GAX** | CTTTATCAATACATACTACAATCAGGGCAICTAAAGGAAGCTCTATTAGATACAGGAGCAGATGA | PR | LUA-002 |
| **STBPR-054-ATX** | CAATTCAAATCACAATAATCAATCGAAIATGGAAACCAAAAATGATAGGGGGAATTGGAGGTTTTAT | PR | LUA-005 |
| **STBPR-054-GTX** | TCAACAATCTTTTACAATCAAATCGAAIATGGAAACCAAAAATGATAGGGGGAATTGGAGGTTTTGT | PR | LUA-006 |
| **STBPR-082-GCX** | TAATCTTCTATATCAACATCTTACGAAATUTGUGGACAUAAAGCTATAGGTACAGTITTAITAGGACCTACACCTGC | PR | LUA-009 |
| **STBPR-082-GTX** | AATCCTTTTACATTCATTACTTACGAAATUTGUGGACAUAAAGCTATAGGTACAGTITTAITAGGACCTACACCTGT | PR | LUA-008 |
| **STBPR-084-ATX** | TACAAATCATCAATCACTTTAATCGACATAAAGCTATAGGTACAGTATTAITAGGACCTACACCTGTCAACAT | PR | LUA-011 |
| **STBPR-084-TGX** | TACACTTTCTTTCTTTCTTTCTTTGACATAAAGCTATAGGTACAGTATTAITAGGACCTACACCTGTCAACTG | PR | LUA-012 |
| **STBPR-088-AAX** | CTACTATACATCTTACTATACTTTAGCTATAGGTACAGTITTAITAGGACCTACACCTGTCAACATAATTGGAAGAAA | PR | LUA-014 |
| **STBPR-088-AGX** | ATACTTCATTCATTCATCAATTCAAGCTATAGGTACAGTITTAITAGGACCTACACCTGTCAACATAATTGGAAGAAG | PR | LUA-015 |
| **STBRT-065-AAX** | CTTTAATCTCAATCAATACAAATCGGCCTGAAAATCCATAUAATACTCCAGTATTTGCUATAAAGAA | RT | LUA-001 |
| **STBRT-065-AGX** | TACATTACCAATAATCTTCAAATCGGCCTGAAAATCCATAUAATACTCCAGTATTTGCUATAAAGAG | RT | LUA-004 |
| **STBRT-074-GXX** | ATCATACATACATACAAATCTACACAGTATTTGCCATAAAGAAAAAAGACAGTACTAAATGGAGAAAAG | RT | LUA-010 |
| **STBRT-074-TXX** | CAATTCATTTACCAATTTACCAATCAGTATTTGCCATAAAGAAAAAAGACAGTACTAAATGGAGAAAAT | RT | LUA-007 |
| **STBRT-075-ACX** | AATCAATCTTCATTCAAATCATCAAGTATTTGCCATAAAGAAAAAAGACAGTACTAAATGGAGAAAATTAAC | RT | LUA-016 |
| **STBRT-075-GTX** | CAATAAACTATACTTCTTCACTAAAGTATTTGCCATAAAGAAAAAAGACAGTACTAAATGGAGAAAATTAGT | RT | LUA-013 |
| **STBRT-103-AAA** | TCAATCAATTACTTACTCAAATACCACATCCUGCAGGGTTAAAAAAGAAA | RT | LUA-019 |
| **STBRT-103-AAC** | AATCCTTTTTACTCAATTCAATCACACATCCUGCAGGGTTAAAAAAGAAC | RT | LUA-022 |
| **STBRT-151-ATX** | CTACAAACAAACAAACATTATCAAGAGACACCAGGIATTAGATATCAGTACAATGTGCTTCCAAT | RT | LUA-028 |
| **STBRT-151-CAX** | CTTTTCAATTACTTCAAATCTTCAGAGACACCAGGIATTAGATATCAGTACAATGTGCTTCCACA | RT | LUA-025 |
| **STBRT-181-TAX** | TTCACTTTTCAATCAACTTTAATCGACAAAAATCTTAGAGCCTTTTAGAAAACAAAATCCAGAUATAGTTATCTA | RT | LUA-031 |
| **STBRT-181-TGX** | TCATTCATATACATACCAATTCATGACAAAAATCTTAGAGCCTTTTAGAAAACAAAATCCAGAUATAGTTATCTG | RT | LUA-034 |
| **STBRT-184-AXX** | CTTTTCATCTTTTCATCTTTCAATAGAGCCTTTTAGAAAACAAAATCCAGAUATAGTTATCTATCAATACA | RT | LUA-037 |
| **STBRT-184-GXX** | CTTTCTACATTATTCACAACATTAAGAGCCTTTTAGAAAACAAAATCCAGAUATAGTTATCTATCAATACG | RT | LUA-040 |
| **STBRT-188-TAT** | TACATCAACAATTCATTCAATACACTTTTAGAAAACAAAATCCAGAUATAGTTATCTATCAATACATGGATGATTTGTAT | RT | LUA-046 |
| **STBRT-188-TTA** | CTTTCAATTACAATACTCATTACACTTTTAGAAAACAAAATCCAGAUATAGTTATCTATCAATACATGGATGATTTGTTA | RT | LUA-043 |
| **STBRT-190-GCX** | TCAATCATCTTTATACTTCACAATAAATCCAGAUATAGTTATCTATCAATACATGGATGAUTTGTATGTAGC | RT | LUA-052 |
| **STBRT-190-GGX** | TCATCAATCTTTCAATTTACTTACAAATCCAGAUATAGTTATCTATCAATACATGGATGAUTTGTATGTAGG | RT | LUA-049 |
| **STBRT-215-ACX** | TATATACACTTCTCAATAACTAACCATAGAACAAAAATAGAGGAACTGAGACAACATCTGTTGAIGTGGGGITTTAC | RT | LUA-055 |
| **STBRT-215-TAX** | CTACTAATTCATTAACATTACTACCATAGAACAAAAATAGAGGAACTGAGACAACATCTGTTGAIGTGGGGITTTTA | RT | LUA-058 |
