# Supplementary material for: DNA Suspension Arrays: Silencing Discrete Artifacts for High-Sensitivity Applications
Source: PLoS One. 2010 Nov 8;5(11):e15476. doi: 10.1371/journal.pone.0015476 (PMC2975679; doi:10.1371/journal.pone.0015476)
Supplement: Table S2 — Reporter capture oligonucleotides. “p-” and “-b” depict 5′-phosphate and 3′-biotin modifications, respectively. (DOC) [file pone.0015476.s007.doc]

**Table S2: Reporter Capture Oligonucleotides (RCOs)**

| **Name** | **Sequence** | **Region** |
| --- | --- | --- |
| **STBIN-148-XXA** | p-AGGAGTAGTAGAATCTATGAATAAAGAATTAAAGAAAATTATAGGACAIGTAAGAGATCAGG-b | IN |
| **STBIN-148-XXC** | p-CGGAGTAGTAGAATCTATGAATAAAGAATTAAAGAAAATTATAGGACAIGTAAGAGATCAGG-b | IN |
| **STBIN-148-XXT** | p-TGGAGTAGTAGAATCTATGAATAAAGAATTAAAGAAAATTATAGGACAIGTAAGAGATCAGG-b | IN |
| **STBIN-155-XXT** | p-TAAAGAATTAAAGAAAATTATAGGACAIGTAAGAGATCAGGCTGAACATCTTAAIACAGCAG-b | IN |
| **STBPR-030-XXT** | p-TACAGTATTAGAAGAUATGAATTTGCCAGGAAIATGGAAACCAAAAATGATAG-b | PR |
| **STBPR-054-XXC** | p-CAAAGTAAGACAGTATGATCAGATACUUITAGAAATUTGTGGACATAAAGC-b | PR |
| **STBPR-082-XXC** | p-CAACATAATTGGAAGAAATCTGTTGACTCAGUTTGG-b | PR |
| **STBPR-084-XXA** | p-AATTGGAAGAAATCTGTTGACTCAGUTTGGUTGUAC-b | PR |
| **STBPR-084-XXT** | p-TATTGGAAGAAATCTGTTGACTCAGUTTGGUTGUAC-b | PR |
| **STBPR-088-XXT** | p-TCTGTTGACTCAGUTTGGUTGCACTTTAAATTTT-b | PR |
| **STBRT-103-XXX** | p-AAATCAGTAACAGTACTGGATGTGGGTGATG-b | RT |
| **STBRT-151-XXG** | p-GGGATGGAAAGGATCACCAGCAATATTCC-b | RT |
| **STBRT-181-XXT** | p-TCAATACATGGATGAUTTGTATGTAGGATCTGACTTAGAAATAG-b | RT |
| **STBRT-184-XTG** | p-TGGATGATTTGTATGTAGGATCTGACTTAGAAATAGGICAGCATAGA-b | RT |
| **STBRT-188-XXX** | p-GTAGGATCTGACTTAGAAATAGGICAGCATAGAACAAAAATAGAGGAAC-b | RT |
| **STBRT-190-XXA** | p-ATCTGACTTAGAAATAGGICAGCATAGAACAAAAATAGAGGAACTGAGAC-b | RT |
| **STBRT-215-XXC** | p-CACACCAGAUAAAAAACATCAGAAAGAACCUCCATT-b | RT |
| **STBRT-065-XXA** | p-AAAAGACAGTACTAAATGGAGAAAATTAGTAGATTTCAGAGAACTTAATAAIAGAACTCAAG-b | RT |
| **STBRT-074-XTA** | p-TAGTAGATTTCAGAGAACTTAATAAIAGAACTCAAGACTTCTGGGAAGTTC-b | RT |
| **STBRT-075-XXA** | p-AGATTTCAGAGAACTTAATAAIAGAACTCAAGACTTCTGGGAAGTTCAATTAG-b | RT |

“p-“ and “-b” depict 5’-phosphate and 3’-biotin modifications, respectively.
