# Supplementary material for: DNA Suspension Arrays: Silencing Discrete Artifacts for High-Sensitivity Applications
Source: PLoS One. 2010 Nov 8;5(11):e15476. doi: 10.1371/journal.pone.0015476 (PMC2975679; doi:10.1371/journal.pone.0015476)
Supplement: Table S3 — Competimer oligonucleotides. (DOC) [file pone.0015476.s008.doc]

**Table S3**: Competimer Oligonucleotides

| **Competimer** | **Sequence** | **Region** |
| --- | --- | --- |
| **PR 030 CO** | GGAGCAGATGATACAGTATTAGAAGAMATRRRTTTRCCAGGAARATGGAAACCAAAAATGATAGGGGGAATTGG | PR |
| **PR 054 CO** | GGAGGTTTTATCAAAGTAAGACAGTATGATCAGRTACYYRTAGAAATYTGYGGACAYAAAGCTATAGGTACA | PR |
| **PR 082 CO** | CCTACACCTGTCAACATAATTGGAAGAAATCTGTTGACTCAGMTTGGYTGYACTTTAAATTTT | PR |
| **PR 084 CO** | ACATAATTGGAAGAAATCTGTTGACTCAGMTTGGYTGYACTTTAAATTTT | PR |
| **PR 088 CO** | GAAATCTGTTGACTCAGMTTGGYTGYACTTTAAATTTTCCCATTAGTC | PR |
| **RT 065 CO** | CYATAAAGAAAAARGACAGTACTAAATGGAGAAAATTAGTAGATTTYAGAGAACTTAATAARARAACTCAAGACTTYTGGGA | RT |
| **RT 074 CO** | TGGAGAAAATTAGTAGATTTYAGAGAACTTAATAARARAACTCAAGACTTYTGGGAAGTTCAATTAGGAAT | RT |
| **RT 075 CO** | TAGTAGATTTYAGAGAACTTAATAARARAACTCAAGACTTYTGGGAAGTTCAATTAGGAATACCACA | RT |
| **RT 103 CO** | AGAAAAAATCAGTAACAGTACTGGATGTGGGTGATGCATAYTTYTC | RT |
| **RT 151 CO** | GGATGGAAAGGATCACCAGCAATATTCCAAWGTAGCATGACAAAAATCTTAGAGCC | RT |
| **RT 181 CO** | GTTATCTATCAATACATGGATGAYTTGTATGTAGGATCTGACTTAGAAATAGRRCAGCATAGARYAAAAATAGAGG | RT |
| **RT 188 CO** | TGGATGAYTTGTATGTAGGATCTGACTTAGAAATAGRRCAGCATAGARYAAAAATAGAGGAACTGAGASARCA | RT |
| **RT 190 CO** | TAGGATCTGACTTAGAAATAGRRCAGCATAGARYAAAAATAGAGGAACTGAGASARCATCTGTT | RT |
| **RT 215 CO** | TTACCACACCAGAYAAAAAACATCAGAAAGAACCYCCATTYCTTTGGATG | RT |
| **IN 148 CO** | GTCAAGGAGTARTAGAATCTATGAATAAAGAATTAAAGAAAATTATAGGACARGTAAGAGATCAGGCTGAACATCT | IN |
| **IN 155 CO** | TARTAGAATCTATGAATAAAGAATTAAAGAAAATTATAGGACARGTAAGAGATCAGGCTGAACATCTTAARACAGCAGTACAAATGGC | IN |
