# Supplementary material for: DNA Suspension Arrays: Silencing Discrete Artifacts for High-Sensitivity Applications
Source: PLoS One. 2010 Nov 8;5(11):e15476. doi: 10.1371/journal.pone.0015476 (PMC2975679; doi:10.1371/journal.pone.0015476)
Supplement: Table S4 — Duplex nucleator oligonucleotides. (DOC) [file pone.0015476.s009.doc]

**Table S4: Duplex nucleator oligonucleotides.**

| **Name** | **Sequence** | **Region** |
| --- | --- | --- |
|  |  |  |
|  |  |  |
| **STBPR-030-RD1** | NATCTGCTCCTGTATCTAATAGAGCTTCCTTTAGCTGCCC | PR |
| **STBPR-054-RD1** | NAAAACCTCCAATTCCCCCTATCATTTTTGGTTTCCATCTTC | PR |
| **STBPR-082-RD1** | NAGGTGTAGGTCCTACTAACACTGTACCTATAGCTTTATGTCCACAAATTTC | PR |
| **STBPR-084-RD1** | NGTTGACAGGTGTAGGTCCTACTAATACTGTACCTATAGCTTTATGTC | PR |
| **STBPR-088-RD1** | NTCTTCCAATTATGTTGACAGGTGTAGGTCCTACTAACACTGTACCTATAGCT | PR |
| **STBRT-065-RD1** | NCTTTATAGCAAATACTGGAGTATTATATGGATTTTCAGGCC | RT |
| **STBRT-074-RD1** | NTTTTCTCCATTTAGTACTGTCTTTTTTCTTTATGGCAAATACTG | RT |
| **STBRT-075-RD1** | NTAATTTTCTCCATTTAGTACTGTCTTTTTTCTTTATGGCAAATACT | RT |
| **STBRT-103-RD1** | NCTTTTTTAACCCTGCAGGATGTG | RT |
| **STBRT-151-RD1** | NTGGAAGCACATTGTACTGATATCTAATCCCTGGTGTCTC | RT |
| **STBRT-181-RD1** | NGATAACTATATCTGGATTTTGTTTTCTAAAAGGCTCTAAGATTTTTGTC | RT |
| **STBRT-184-RD1** | NGTATTGATAGATAACTATATCTGGATTTTGTTTTCTAAAAGGCTCT | RT |
| **STBRT-188-RD1** | NCAAATCATCCATGTATTGATAGATAACTATATCTGGATTTTGTTTTCTAAAAG | RT |
| **STBRT-190-RD1** | NTACATACAAATCATCCATGTATTGATAGATAACTATATCTGGATTT | RT |
| **STBRT-215-RD1** | NAAACCCCCACCTCAACAGATGTTGTCTCAGTTCCTCTATTTTTGTTCTATG | RT |
| **STBIN-148-RD1** | NACTTTGGGGATTGTAGGGAATGCC | IN |
| **STBIN-155-RD1** | NCATAGATTCTACTACTCCTTGACTTTGGGGATTGTAGGGAAT | IN |
