# Supplementary material for: DNA Suspension Arrays: Silencing Discrete Artifacts for High-Sensitivity Applications
Source: PLoS One. 2010 Nov 8;5(11):e15476. doi: 10.1371/journal.pone.0015476 (PMC2975679; doi:10.1371/journal.pone.0015476)
Supplement: Table S5 — PCR Oligonucleotides. (DOC) [file pone.0015476.s010.doc]

**Table S5: PCR Oligonucleotides**

| **Name** | **Oligonucleotide Sequence** |
| --- | --- |
| **ML-gag-F3** | GGACAYCAAATGAAAGATTGYACTGARAGACAGGC |
| **ML-int-R2** | CCCCCCTTTTCTTTTAAAATTGTGRATGAATACTGCC |
| **RTS2** | TCAAAAATTGGGCCTGAAAATCCAT |
| **RTA8** | GCTATTAAGTCTTTTGATGGGTCAT |
| **LigPol-F2** | GGGCAGCTAAAGGAAGC |
| **LigPol-R2** | CTGCTGTCTTAAGATGTTCAGC |
