# Supplementary material for: DNA Suspension Arrays: Silencing Discrete Artifacts for High-Sensitivity Applications
Source: PLoS One. 2010 Nov 8;5(11):e15476. doi: 10.1371/journal.pone.0015476 (PMC2975679; doi:10.1371/journal.pone.0015476)
Supplement: Table S6 — Assembly and junction span oligonucleotides for ligase-mediated oligonucleotide assembly. “p-” and “-amine” depict 5′-phosphate and 3′-amine modifications, respectively. (DOC) [file pone.0015476.s011.doc]

**Table S6: Assembly and Junction Span Oligonucleotides for Ligase-Mediated Oligonucleotide Assembly**

| **Name** | **Sequence** |
| --- | --- |
| **AP01** | p-GGGCAGCTAAAGGAAGCTCTATTAGATACAGGAGCAGATRATACAGTATTAGAAGATATGAATTTGCCAGGAAGATGGAA |
| **AP02** | p-ACCAAAAATGATAGGGGGAATTGGAGGTTTTRTCAAAGTAAGACAGTATGATCAGATACCCATAGAAATTTGTGGACATA |
| **AP03** | p-AAGCTATAGGTACAGTATTAGTAGGACCTACACCTGYCAACWKWATTGGAAGAARTCTGTTGACTCAGTTTGGTTGCACT |
| **AP04** | p-TTAAATTTTGGCCTGAAAATCCATATAATACTCCAGTATTTGCCATAAAGARAAAAGACAGTACTAAATGGAGAAAAKTA |
| **AP05** | p-RYAGATTTCAGAGAACTTAATAAGAGAACTCAAGACTTCTGGGAAGTTCAATTAGGAATACCACATCCTGCAGGGTTAAA |
| **AP06** | p-AAAGAAMAAATCAGTAACAGTACTGGATGTGGGTGATGGAGACACCAGGGATTAGATATCAGTACAATGTGCTTCCAMWG |
| **AP07** | p-GGATGGAAAGGATCACCAGCAATATTCCAAAGTAGCATGACAAAAATCTTAGAGCCTTTTAGAAAACAAAATCCAGATATA |
| **AP08** | p-GTTATCTRTCAATACRTGGATGATTTGTWWGTAGSATCTGACTTAGAAATAGGGCAGCATAGAACAAAAATAGAGGAAC |
| **AP09** | p-TGAGACAACATCTGTTGAGGTGGGGGTTTWMCACACCAGATAAAAAACATCAGAAAGAACCTCCATTGGCATTCCCTACA |
| **AP10** | p-ATCCCCAAAGTMRHGGAGTAGTAGAATCTATGMATAAAGAATTAAAGAAAATTATAGGACAGGTAAGAGATCAGGCTGAACATCTTAAGACAGCAG |
| **JS01** | CCCCCTATCATTTTTGGTTTCCATCTTCCTGGCA-amine |
| **JS02** | CTAATACTGTACCTATAGCTTTATGTCCACAAATTTCTATG-amine |
| **JS03** | ATTTTCAGGCCAAAATTTAAAGTGCAACCAAACTGAG-amine |
| **JS04** | TTATTAAGTTCTCTGAAATCTRYTAMTTTTCTCCATTTAGTACTG-amine |
| **JS05** | ACTGTTACTGATTTKTTCTTTTTTAACCCTGCAGGAT-amine |
| **JS06** | GGTGATCCTTTCCATCCCWKTGGAAGCACATTGTAC-amine |
| **JS07** | ATCCAYGTATTGAYAGATAACTATATCTGGATTTTGTTTTCT-amine |
| **JS08** | TCAACAGATGTTGTCTCAGTTCCTCTATTTTTGTTCTATG-amine |
| **JS09** | TCCDYKACTTTGGGGATTGTAGGGAATGCCAATG-amine |

“p-“ and “-amine” depict 5’-phosphate and 3’-amine modifications, respectively.
