# Supplementary material for: DNA Suspension Arrays: Silencing Discrete Artifacts for High-Sensitivity Applications
Source: PLoS One. 2010 Nov 8;5(11):e15476. doi: 10.1371/journal.pone.0015476 (PMC2975679; doi:10.1371/journal.pone.0015476)
Supplement: Table S7 — Synthetic HIV-1 subtype B consensus clone sequences. (DOC) [file pone.0015476.s012.doc]

**Table S7**: Synthetic HIV-1 subtype B consensus clone sequences

|  | **Clone** | | | | | |
| --- | --- | --- | --- | --- | --- | --- |
| **Codon** | **LP02** | **LP10** | **LP19** | **LP21** | **LP38** | **LP40** |
| **PR 030** | GAT | GAT | AAT | AAT | GAT | AAT |
| **PR 054** | GTC | ATC | GTC | GTC | GTC | ATC |
| **PR 082** | GTC | GCC | GTC | GTC | GTC | GTC |
| **PR 084** | ATA | TGT | ATT | ATA | ATT | ATT |
| **PR 088** | AGT | AAT | AGT | AGT | AGT | AAT |
| **RT 065** | AGA | AAA | AAA | AAA | AGA | AGA |
| **RT 074** | TTA | GTA | GTA | GTA | TTA | TTA |
| **RT 075** | GTA | ACA | ACA | GTA | ACA | ACA |
| **RT 103** | AAC | AAA | AAA | AAA | AAA | AAA |
| **RT 151** | ATG | ATG | CAG | ATG | CAG | CAG |
| **RT 181** | TAT | TGT | TGT | TAT | TAT | TAT |
| **RT 184** | GTG | GTG | ATG | ATG | ATG | GTG |
| **RT 188** | TAT | TTA | TAT | TAT | TTA | TAT |
| **RT 190** | GGA | GGA | GGA | GGA | GCA | GCA |
| **RT 215** | ACC | ACC | ACC | ACC | ACC | ACC |
| **IN 148** | AAC | AAT | AAA | CAT | CAT | AAC |
| **IN 155** | CAT | AAT | AAT | AAT | AAT | AAT |
